# Comprehensive Immunolocalization Studies of a Putative Serotonin Receptor from the Alimentary Canal of *Aedes aegypti* Larvae Suggest Its Diverse Roles in Digestion and Homeostasis

**DOI:** 10.1371/journal.pone.0146587

**Published:** 2016-01-25

**Authors:** Adelina Petrova, David Franklin Moffett

**Affiliations:** School of Biological Sciences, Washington State University, Pullman, WA, United States of America; Rosalind Franklin University, UNITED STATES

## Abstract

Serotonin regulates key processes including digestion and homeostasis in insects. Serotonin effects are mediated by serotonin receptors that transduce information through initiation of second messenger signaling pathways. Lack of information on serotonin receptors associated with the alimentary canal impedes the understanding of the serotonergic role in insect physiology. To address this void, the present study has cloned and identified a putative serotonin receptor (hereafter AaSeR-1) from the alimentary canal of *Aedes aegypti* (yellow fever mosquito) larvae. In addition to *in-silico* analyses of AaSeR-1 primary sequence, immunohistochemical investigations were carried out to elucidate receptor expression patterns. Specific AaSeR-1 immunofluorescence was detected in the caeca, the mid- and hindgut, including the Malpighian tubules. These findings point out not only receptor ubiquitous nature but also its involvement in regulation of different stages of nutrient processing and homeostasis. Furthermore, AaSeR-1 may mediate an array of effects through its differential expression at various cell compartments. While AaSeR-1 specific immunofluorescence was depicted in the nucleus and nucleolus of principal cells of the anterior midgut, in the posterior, analyses suggest receptor association with the plasma membrane of both principal and regenerative cells. In addition, AaSeR-1 immunofluorescence was also found in some enteroendocrine cells and in both circular and longitudinal muscles that innervate the alimentary canal. Overall, immunohistochemical analyses of AaSeR-1 expression indicate that this receptor exercises multiple roles in digestion- and homeostasis-related mechanisms.

## Introduction

Serotonin (5-hydroxytryptamine; hereafter 5-HT) is a biogenic amine with a long evolutionary history in both invertebrate and vertebrate phyla. In vertebrate systems, 5-HT affects several behavioral processes such as appetite, sleep, circadian clock, and has different neuroendocrine functions impacting (directly or indirectly) almost every physiological process [1–4]. Several disorders such as depression, schizophrenia, obsessive-compulsive disorder, suicidal behavior, migraine and many others are treated by targeting the serotonergic network [5]. 5-HT is distributed in the nervous system as well as in many peripheral tissues. Although 5-HT is associated with the brain and the nervous system, ~90% of its production in humans is carried out by the gut enterochromaffin cells to modulate gastrointestinal (GI) tract motility [4].

In contrast to vertebrates, knowledge of 5-HT roles in insects is limited. However, studies to date have underlined the importance of this molecule in insect physiology and homeostasis. For instance, in addition to its role as a neurotransmitter, 5-HT has neurohormone activity to coordinate diverse physiological functions in the salivary glands [6], the Malphigian tubules [7–8] and the circulatory system [9–10]. Evidence for serotonergic regulation of digestion-related processes such as gut motility, secretion of alkali in the anterior and acid in the posterior midgut, as well as nutrient absorption in the anterior midgut of mosquito larvae has been provided [11–15]. In addition, 5-HT affects many insect behavioral processes such as aggression [16] and gregarization of locusts [17], as well as learning and memory in fruit flies (*Drosophila melanogaster*) [18] and honeybees (*Apis mellifera)* [19].

5-HT mediates its diverse effects through an array of receptors that are part of the G protein-coupled receptor (hereafter GPCR) group. Several conserved features such as seven transmembrane domains (hereafter TM domains), an extracellular N-terminal and a cytoplasmic C-terminal region are common for this group of receptors [20, 21]. Serotonergic effects are transduced by signaling cascades initiated by ligand binding. This results in receptor conformational changes enabling G protein coupling and commencement of second messenger signaling pathways (cyclic adenosine monophosphate (hereafter cAMP), inositol trisphosphate (hereafter IP_3_) [20, 21]. Expression patterns, primary sequence, activation mechanisms, etc. are some of the key features determining 5-HT receptors functional diversity and pharmacological properties. Based on agonist/antagonist studies, to date, serotonergic receptors of vertebrates are classified into seven pharmacologically distinct groups (5-HT_1, 2, 3, 4, 5, 6, 7_) and several subgroups [22, 23]. Although less comprehensive, such categorization is also available for insect 5-HT receptors (5-HT_1, 2, 7_) [24, 25].

Insect gut is a prominent organ whose primary functions are digestion and absorption of nutrients as well as maintaining ion and water homeostasis. These roles define the gut as the ‘powerhouse’ of the insect body whose functions affect all other physiological processes at multiple levels (cellular, tissue, whole body). Although several 5-HT receptors from insects have been cloned and characterized [26–30], only a few are known for the mosquito gut [31]. To address this void, the current study has initiated investigations on a putative 5-HT receptor in *Aedes aegypti* (yellow fever mosquito) larval alimentary canal. The importance of this mosquito species to human health cannot be understated; it is a vector for several pathogens that are serious threats to human health (dengue fever, chikungunya and yellow fever). Dengue alone is endemic in over 100 nations around the world, with an estimated 50–100 million infected individuals each year (http://www.who.int/mediacentre/factsheets/fs117/en/). Many facts, such as almost half the world’s population being at risk of contracting mosquito-borne diseases, as well as a lack of effective drugs to control them, underline the urgent need for the development of effective technologies for mosquito control. Since research to date indicates that 5-HT mediates key physiological processes, generating knowledge of how the serotonergic system regulates digestion and homeostasis in mosquitoes may provide a basis for the development of such approaches. Furthermore, insects are useful systems for addressing fundamental conserved mechanisms in cell signaling and function.

The current study is part of a large platform of investigations in which the overall objective is to elucidate the physiological role of the serotonergic system in arthropod digestion and homeostasis. This research aimed to provide specific answers as well as establishing a basis for further analyses. AaSeR-1 primary sequence and receptor distribution profile were at the core of the initial investigations. Results have not only provided valuable information concerning this gene, but have also generated reasonable evidence to reexamine the role of 5-HT as a neurohormone in mosquitoes and other arthropod systems. The presence of AaSeR-1 at multiple sites in the alimentary canal of *Aedes aegypti* larvae suggests that this receptor has diverse functions in mosquito digestion and homeostasis.

## Materials and Methods

### Insects

Eggs of *Aedes aegypti* (Vero Beach strain; hereafter *Aedes*) originally provided by Dr. Marc Klowden, University of Idaho, were hatched in a 50:50 mixture of tap and deionized water to which a small amount of baker’s yeast was added to provide anoxic conditions. Larvae were maintained in 50:50 mixture of tap and deionized water at 27–30°C and fed each day with ground fish food (TetraColor).

### RNA extraction and quantification

For total RNA extraction, larval guts (3^rd^ - 4^th^ instar) were dissected in cold 1 X PBS (137 mM NaCl, 10 mM Na_2_HPO_4_, 1.8 mM KH_2_PO_4_, 2.7 mM KCl, pH 7.4; all purchased from J. T. Baker) and immediately placed in TRI Reagent (Sigma). About 30 larval guts were homogenized and RNA isolation procedures were carried out following manufacturer’s protocol.

### Identification of PCR fragments encoding *AaSeR-1*

First-strand cDNA was obtained by reverse transcription of total RNA (2 μg) with oligo dT universal primer. The reaction was carried out with Reverse transcriptase III (LifeTechnologies) following manufacturer’s protocol. Specific primers were designed by analyzing the predicted sequence of a putative 5-HT receptor with accession number AAEL007644 (https://www.vectorbase.org/organisms/aedes-aegypti). To amplify regions of *AaSeR-1*, a PCR containing 1 x High Fidelity PCR Buffer (LifeTechnologies), 200 μM dNTPs (Fermentas), 1.6 mM MgSO_4_ (LifeTechnologies), 1 μM of each primer (forward primer 5’-TGTATCCACCACCATTGTATCTGTGTACA-3’, and reverse primer 5’-CTCAGCTGCCACAGGAGCACTAGCA-3’), 0.2 units of Platinum High Fidelity *Taq* polymerase (LifeTechnologies) and 4 μl first strand cDNA in total volume of 40 μl, was carried out. cDNA fragments were amplified under the following conditions: one cycle at 94°C for 180 s, followed by 44 cycles at 94°C for 30 s, 55°C for 60 s, 68°C for 90 s; and one final cycle at 68°C for 10 min. The amplified products were analyzed by agarose gel electrophoresis; 30 μl of each reaction was loaded on a 0.8% agarose gel and run at constant voltage 0.5 V/cm. PCR fragments were visualized under UV illumination using a Gel Logic 200 Imaging System (Kodak). DNA purification from the excised bands of the agarose gel was carried out by using a Qiaex^®^ II gel extraction kit (Qiagen). The purified product was ligated into pCR^®^2.1-TOPO and transformed into TOP10 chemically competent cells of *E*. *coli* (TOPO Cloning System, LifeTechnologies). The transformed bacteria were plated on agar plates containing 50 μg/ml kanamycin (Fisher Scientific) and 40 μg/ml of 5-bromo-4-chloro-3-indolyl-β-D-galactoside (XGal; Fisher Scientific) for blue/white selection. Ten colonies were randomly selected and grown overnight at 37°C in LB medium with the appropriate antibiotic. Plasmid purification was carried out with QIAprep^®^ Spin Miniprep (250) kit (Qiagen) following the manufacturer’s procedures. Restriction digestion was performed with *EcoRI* (Fermentas). Plasmids containing inserts were sequenced at the WSU sequencing facilities (Center for Reproductive Biology—Molecular Biology and Genomics Core) with M13 reverse primer or T7 promoter primer.

### Completion of *AaSeR-1* cDNA sequence using 5’ and 3’ Rapid Amplification of cDNA Ends (RACE) PCR

To obtain complete cDNA sequence, RACE PCR (Clontech) was carried out as followed: For the 3’ untranslated region (3’UTR), 2 μg of total RNA was reverse transcribed with a specially designed oligo d(T) primer, where an additional anchor sequence was added to the end of the primer (Clontech). A similar reaction was carried out for the 5’UTR, where both oligo d(T) primer (specially designed for 5’ end extension) and SMART oligos were used (Clontech). PCRs were carried out with sequence specific primers and a universal primer (Clontech). The specific primer sequences were as follows: 3’ RACE forward primer 5’-TGTATCCACCACCATTGTATCTGTGTACA-3’ and 5’ RACE reverse primer 5’-ACCAGCATACGCGTCCACCATCCGTA-3’. Gel purification, cloning, transformation and sequencing procedures were the same as described above.

### Identification of cDNA encoding *AaSeR-1*

Nucleotide sequences were edited, assembled in Sequencher 4.8. The resulting sequence was used to query the GenBank database at NCBI (http://blast.ncbi.nlm.nih.gov/Blast.cgi) using BLAST (blastx) sequence comparison algorithms to search for identical sequences, as well as to predict putative gene function.

### Amino acid sequence analyses

The amino acid sequence of AaSeR-1 was analyzed in Expasy (http://web.expasy.org/compute_pi/) to predict some parameters such as isoelectric point (pI) and molecular weight (Mw). Further, AaSeR-1 sequence was subjected to TMHMM Server v.2.0 (http://www.cbs.dtu.dk/services/TMHMM/) and PROSITE (http://prosite.expasy.org/cgi-bin/prosite/ScanView.cgi?scanfile=61772733485.scan.gz) analyses to predict transmembrane regions and reveal signature motifs for GPCRs. Several amino acid sequences from different species along with AaSeR-1 were aligned to identify conserved domains.

### Antiserum production

Bcepred (http://www.imtech.res.in/raghava/bcepred/) was used to predict peptide physicochemical properties (hydrophilicity, flexibility, polarity and exposed surface) and epitope regions. A small amino acid region on the C-terminus (RSNNTSNRSMPTYNSNTSD) was chosen for production of synthetic peptide antigen, which was conjugated with activated Keyhole limpet hemocyanin. Antibodies were produced in rabbit hosts, where seven immunization shots of the conjugated product (0.5mg of antigen each time) were administered over 6 month period. Antigen and antibody production were carried out by YenZym (http://www.yenzym.com/).

### Immunodetection and Immunohistochemical analyses of AaSeR-1

For immunodetection of AaSeR-1, larval guts (3^rd^ - 4^th^ instar) were dissected into cold 1 x PBS and placed in 2% SDS (Sodium dodecyl sulfate; Sigma). Samples were frozen at -20°C overnight to help membrane dissociation. Prior to protein separation, samples were homogenized and incubated at 100°C for 2–3 min, followed by immediate cooling on ice. The clear supernatant containing total protein extract was collected after centrifugation at 13,000 rpm for 10 min at 4°C. Samples (protein extract of ~ 2 guts/lane) were run on 10% sodium dodecyl sulfate polyacrylamide gel to separate proteins by molecular weight using a Laemmli buffer system [32]. After sodium dodecyl sulfate polyacrylamide gel electrophoresis (SDS-PAGE) separation, proteins were transferred on 0.4 μm nitrocellulose membrane (Bio-Rad) using tank-blotting system (Bio-Rad). Prior to blocking, membranes were rinsed several times with distilled water and final wash was carried out with washing buffer (1 X TBST = 137 mM NaCl, 2.7 mM KCl, 19 mM Tris-base (LifeTechnologies), 0.01% Tween 20 (Fisher Scientific)), followed by incubation (1–2 h) in blocking solution (1 X TBST with 5% (w/v) skimmed milk powder). After blocking, membranes were transferred and incubated (4 h) in solution containing either (i) anti-AaSeR-1 antiserum (1:2500) for receptor detection, (ii) pre-immune serum (1:2500) plus anti-KLH antibody (1:10000) for negative control, (iii) peptide pre-absorbed anti-AaSeR-1 antiserum (1:2500) plus anti-KLH antibody (1:10000) for specificity analyses. After several rinses with washing buffer (4 x 10 min), membranes were incubated in secondary antibodies (1:7000; Anti-Rabbit Alkaline Phosphatase; Sigma) for 2 hours. This was followed by a new set of washing steps (4 x 10 min), followed by final wash with reaction buffer (100 mM Tris-HCl, pH 9.5; 100 mM NaCl; 5 mM MgCl_2_). For development, each membrane was incubated into 15 ml of reaction buffer containing 120 μl 5-Bromo-4-chloro-3-indolyl phosphate (BCIP stock solution 20 mg/ml; Thermo Scientific) and 90 μl Nitroblue tetrazolium (NBT stock solution 50 mg/ml; Thermo Scientific). Reactions were carried out in a dark setting and stopped by washing the membranes with distilled water.

For immunolocalization of AaSeR-1, after dissection, larval guts were incubated overnight in 100 mM Phosphate buffer pH 7.4 (100 mM Na4HPO4, 100 mM NaH2PO4; J.T. Baker) containing 4% Paraformaldehyde (Ted Pella Inc). Following fixation, samples were washed (6 x 10 min) with 1 x PBS pH 7.4, and then blocked (16 h) in 1 x PBS (pH 7.4) containing 5% goat serum (Gibco), 0.1% KLH (Sigma) and 5% Triton (Sigma). This was followed by incubation (32–34 h) in antiserum diluent solution (1 x PBS containing 5% goat serum, 0.1% KLH and 0.5% Triton) containing either: (i) anti-AaSeR-1 antiserum (1:500) for receptor detection, (ii) pre-immune serum (1:500) + anti-KLH antibody (1:10000) for negative control, and (iii) pre-absorbed anti-AsSeR-1 antiserum (1:500) + anti-KLH antibody (1:10000) for specificity analyses. The following washing period was extended to 2–3 hours (10 x 12–15 min) to ensure complete removal of unbound primary antibodies. To visualize the specific binding of anti-AaSeR-1 antibodies, samples were incubated overnight at 4°C with Alexa Fluor^®^ 488 Goat Anti-Rabbit IgG (H+L) antibodies (1:300; Molecular Probes-Life technologies). Final wash (1 x PBS, pH 7.4) was carried out for 16–20 hours with minimum of 15 changes of buffer. After the final wash, samples were transferred onto glass slides and immersed into VectaShield (Vector Labs) mounting medium with 4', 6-diamidino-2-phenylindole staining (DAPI; blue color), which is a DNA-specific marker. Imaging analyses were carried out on Zeiss 510 META Confocal Laser Scanning Microscope at the Franceschi Microscopy and Imaging Center, Washington State University, Pullman, WA, USA.

## Results and Discussion

### Cloning and amino acid sequence analyses of *AaSeR-1*

**Fig 1:**
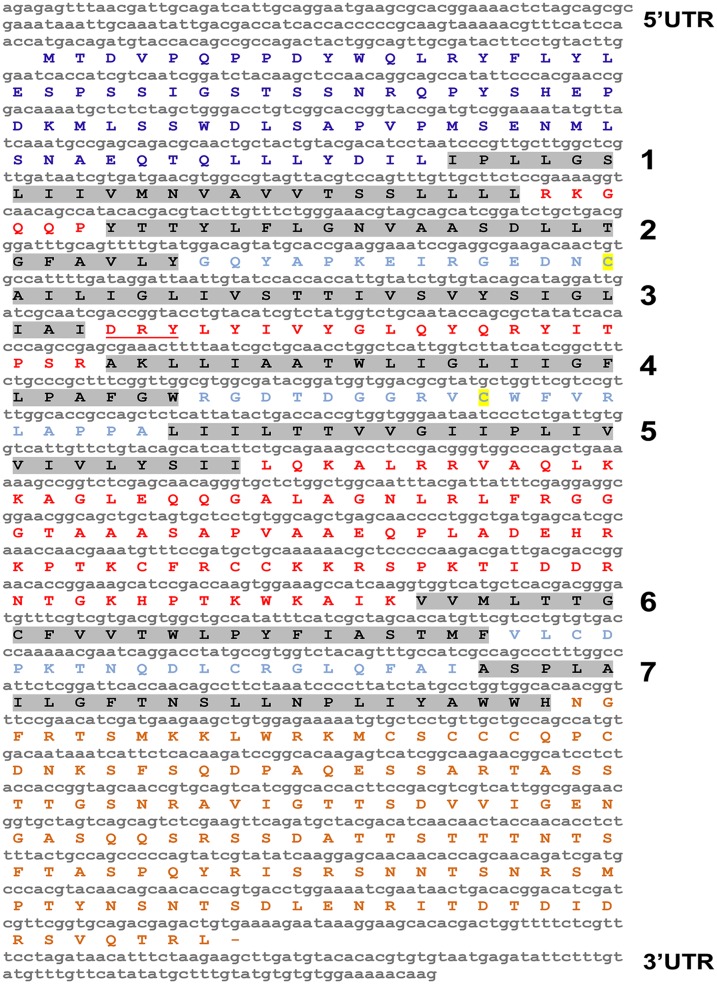
Complete *AaSeR-1* cDNA sequence with predicted ORF and 5’ and 3’ UTR. Prediction analyses identified features typical of GPCRs—seven TM domains (in grey), extracellular N- terminal (in dark blue), cytoplasmic C-terminal (in brown) and six loops (3 extracellular—in pale blue; 3 cytoplasmic—in red). The ‘DRY’ motif (underlined in red) which is highly conserved among GPCRs is positioned in the second cytoplasmic loop.

**Fig 2:**
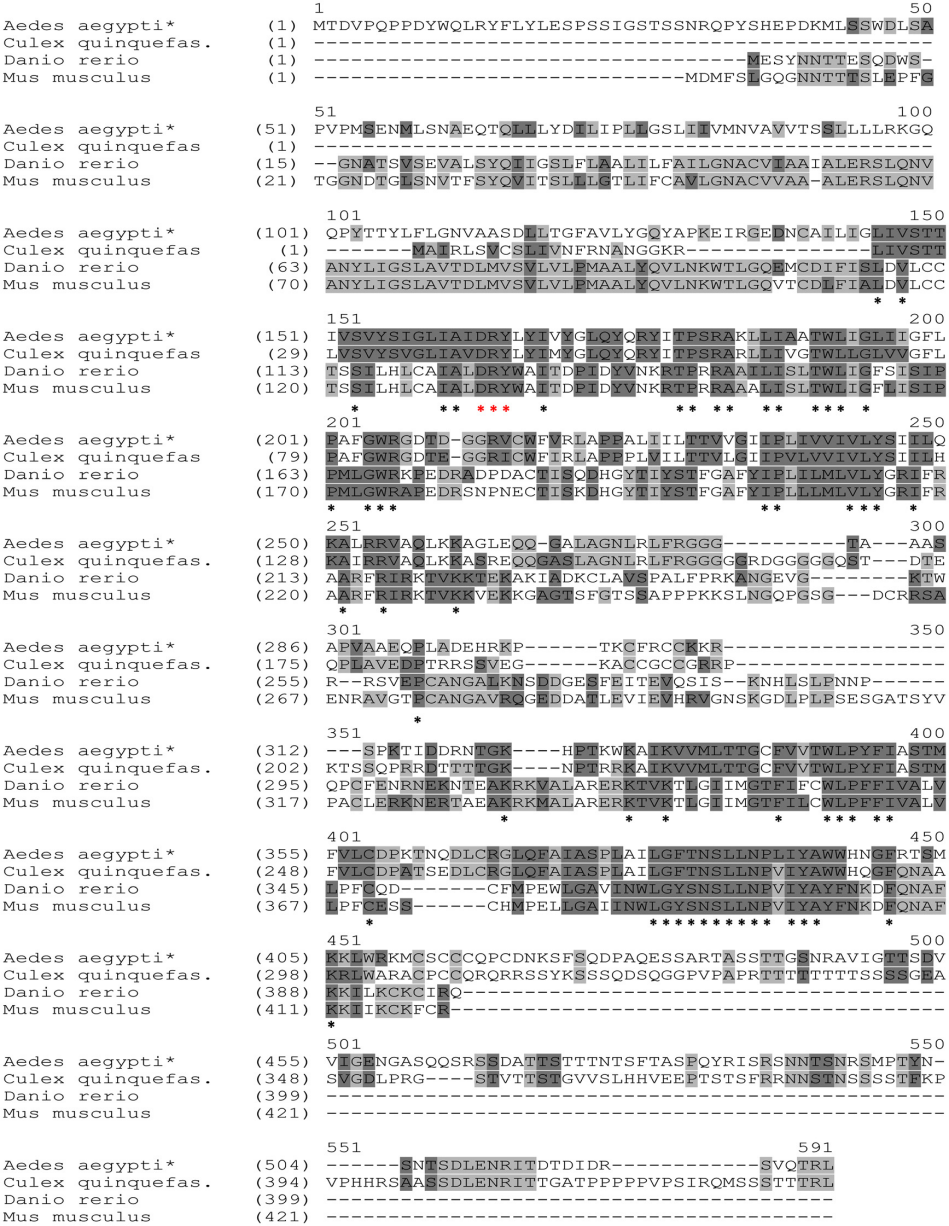
Alignment of AaSeR-1 (*Aedes aegypti*; AIK28864) with amino acid sequences of putative 5-HT receptors from: *Culex quinquenfasciatus* (mosquito; XP_001863625), *Danio rerio* (zebrafish; NP_001116793) and *Mus musculus* (house mouse; AAA81519). Conserved regions are marked with asterisks (‘DRY’ motif in red).

The complete cDNA sequence of *AaSeR-1* consists of 1843 nucleotides with 1581 bp (including stop codon) of a coding sequence and an additional 124 bp of 5’ UTR and 138 bp of 3’ UTR (NCBI, KJ935034). *AaSeR-1* encodes a protein with 526 amino acids (Fig 1) with a predicted molecular mass of ~58 kDa and an isoelectric point of 9.27. Analyses of the AaSeR-1 amino acid sequence have identified specific regions characteristic of GPCRs—seven TM domains (Fig 1, highlighted in gray) connected by extracellular (Fig 1 in pale blue) and intracellular loops (Fig 1 in red). An extracellular N-terminus (Fig 1 in dark blue, 73 amino acids) and cytoplasmic C-terminus (Fig 1 in brown, 129 amino acids), as well as a large cytoplasmic loop between the 5^th^ and 6^th^ TM domains (Fig 1 in red, 85 amino acids) were predicted as well. Further analyses have identified a ‘VSVySIGLIAIDRYLyI’ region typical of family A from the GPCR group (5-HT receptors are part of family A of the GPCRs). These findings corroborate with other Blast (NCBI) results predicting the putative function of AaSeR-1 as a 5-HT receptor. Additional investigations indicated a possible formation of a disulfide bond between cysteine 139 and 215 (Fig 1, highlighted in yellow) that may contribute to receptor stability and function [33, 34]. The ‘DRY’ motif (Asp-Arg-Tyr; 163–165 position; Fig 2, red asterisks) positioned at the second cytoplasmic loop is highly conserved among GPCRs and implicated in receptor activation and signal transduction [35, 36].

Blast analyses confirmed that *AaSeR-1* had the highest identity scores with 5-HT receptors, especially with those from mosquitoes (NCBI, Blastx). To show overall trends, AaSeR-1 (NCBI, AIK28864) was aligned and compared with amino acid sequences from *Culex quinquefasciatus*—mosquito (NCBI, XP_001863625), *Danio rerio—*zebrafish, (NCBI, NP_001116793) and *Mus musculus—*house mouse (NCBI, AAA81519), (Fig 2). While AaSeR-1 exhibited ~49% identity with *Culex* amino acid sequence, only 17–18% of homology was observed with those of zebrafish and mouse. The last TM domain was the only conserved region between analyzed sequences that bears noting (Fig 2). Taken together, these analyses suggest that although the serotonergic signaling has many conserved features, the narrow physiological specialization is reflected in variation of 5-HT receptors primary sequences. Thus, it is likely that sequence variations are one of the key factors determining receptor functional characteristics.

### Specificity and Functional expression analyses

**Fig 3:**
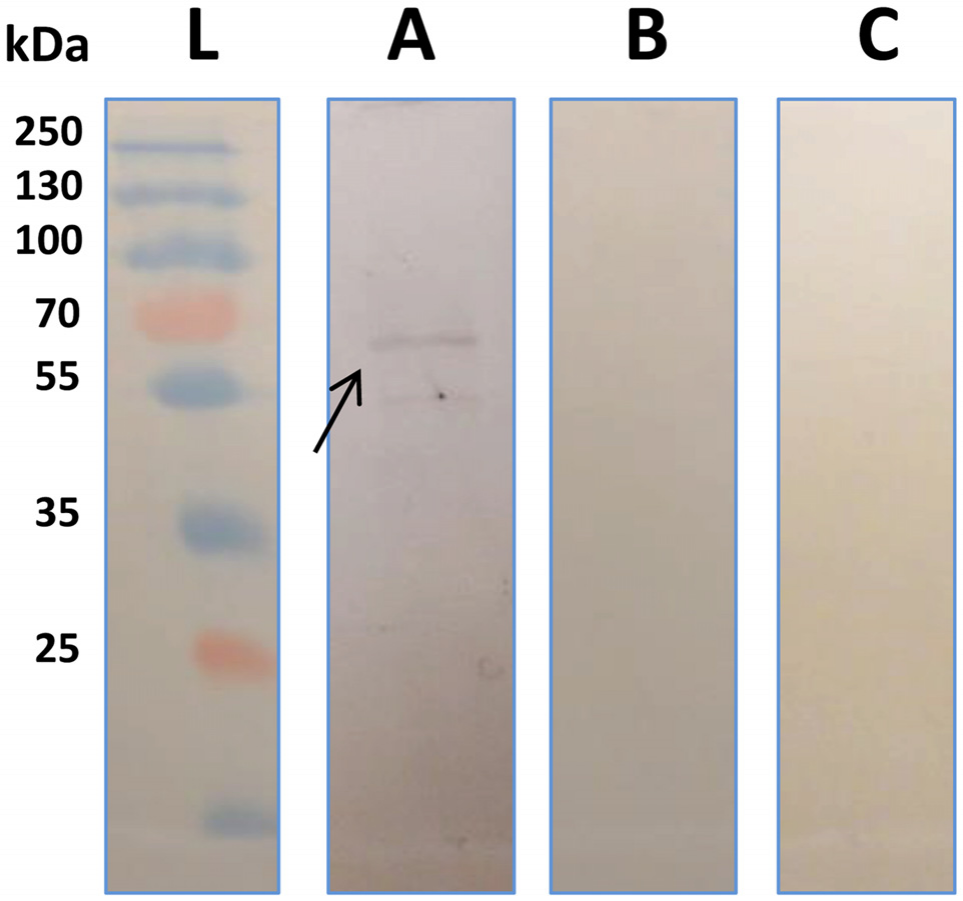
Western blotting analyses of AaSeR-1. Following extraction, protein extracts from *Aedes* larval guts were run on 10% SDS-PAGE gel in Laemmli buffer system. After separation, proteins were transferred on nitrocellulose membranes (0.45 μm) and probed with (A) anti-AaSeR-1 antiserum (1:2500), (B) pre-immune serum (1:2500) + anti-KLH antibodies (1:10000), and (C) peptide pre-absorbed anti-AaSeR-1 antiserum + anti-tKLH antibodies (1:10000). Primary antibody binding was visualized with anti-Rabbit Alkaline Phosphatase (1:7000). The arrow is depicting immunoreactive gut protein of ~60 kDa which is in agreement with a predicted size of AaSeR-1 (~58 kDa).

Anti-AaSeR-1 antiserum (1:2500) had high affinity to a protein with molecular weight of ~60 kDa (Fig 3A), which is in agreement with the predicted size of AaSeR-1 (~58 kDa). A weaker band visible at the 55 kDA mark indicated presence of either a splice variant or degradation product. Protein extracts from *Aedes* larval guts that were subjected to negative controls ((i) pre-immune serum (rabbit) with added anti-KLH antibodies (Fig 3B) and (ii) peptide pre-absorbed antiserum (Fig 3C)), did not yield any bands. While the first suggested that there is no cross-reactivity between mosquito endogenous proteins and host pre-immune serum, including anti-KLH antibodies, the second indicated that anti-AaSeR-1 antiserum is highly specific and binds to its target, the endogenous AaSeR-1 in *Aedes* larval gut.

**Fig 4:**
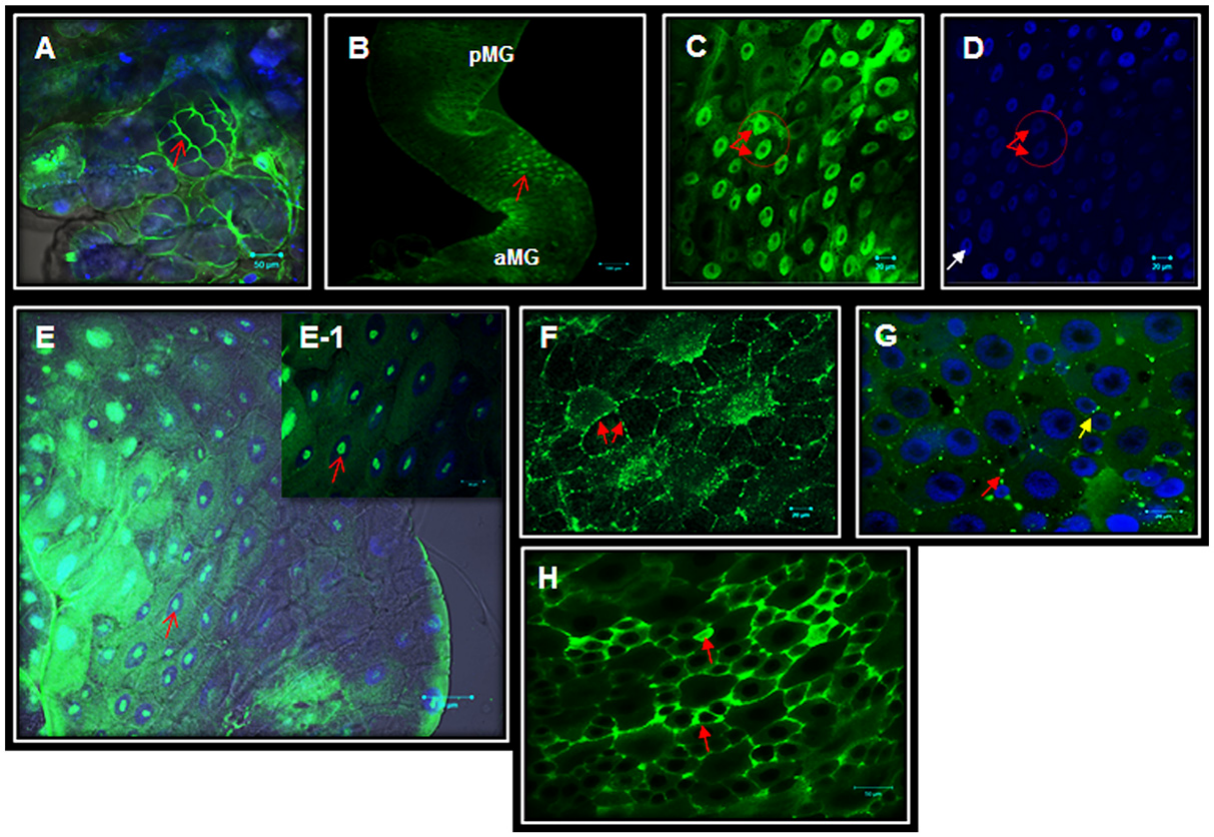
AaSeR-1 expression in the caeca and the midgut of *Aedes* larvae. AaSeR-1 specific immunofluorescence (in green; Ex. 488) was depicted in some caecal cells (A; a red arrow showing an example of AaSeR-1 specific immunofluorescence, hereafter red arrow). Low (B, red arrow) and high (C; red arrow) magnification images of the anterior midgut (aMG) are showing AaSeR-1 expression in the nucleus of some principal cells (in green). Co-localization analyses with DAPI (D; in blue, Ex. 405), which is a DNA-specific fluorescent dye, permitted validation of AaSeR-1 nuclear localization. Usually, DAPI does not stain nucleoli (D; a white arrow showing an unstained nucleolus). Therefore, this enabled the differentiation of AaSeR-1 expression in the nucleoli (E, E-1) of some principal cells of the aMG. In the posterior midgut (pMG), AaSeR-1 immunofluorescence profile suggest receptor association with the plasma membrane of principal (F; red arrow) and regenerative cells (G; yellow arrow). AaSeR-1 was also found in some EECs (H; red arrow). Scale bars (μM) are included in each image.

Immunolocalization studies have shown that AaSer-1 has a ubiquitous presence in different sections of the alimentary canal of *Aedes* larvae. The alimentary canal has three morphologically and functionally distinct zones–the foregut, the midgut with anterior and posterior areas, and the hindgut [37]. The foregut and the midgut are connected through cardia (cardiac valve) that holds eight pouch-like structures called gastric caeca [37]. The posterior end of the midgut is joined to the hindgut through the pylorus (pyloric valve) from where the Malpighian tubules (hereafter MT) arise [37]. Each zone of the alimentary canal has specific roles in food processing and absorption. The midgut, however, plays a central part [38]. Once ingested, food is digested and absorbed in temporal and spatial patterns [38]. This partitioning is aided by the formation of the peritrophic membrane which envelops ingested food. While larger molecules such as peptides and polymers are broken down in the endoperitrophic space (within the peritrophic membrane), smaller ones (dimers, oligomers) are processed in the ectoperitrophic space (between the peritrophic membrane and the epithelial surface) [38]. Peristaltic movements of the alimentary canal direct food digestion in opposite directions: while food particles in the endoperitrophic space move towards the hindgut, the ectoperitrophic fluid (containing small molecules) with semi-digested nutrients is directed towards the caeca. The caeca in *Aedes* larvae are prominent, consistent with their apparent key roles in digestion and nutrient absorption [39]. AaSeR-1 immunofluorescence associated with some gastric caecal cells (Fig 4A) suggest that this receptor mediates 5-HT effects in the caeca. No specific expression patterns of AaSer-1 were observed that may permit correlation with previously reported cell types in the caeca [39]. The brightfield image of caeca superimposed with green (AaSeR-1-specific, Ex. 488) and blue fluorescence (DNA-specific, Ex. 405) provides a more informative presentation of AaSeR-1 expression patterns in relation to cell compartment and tissue type. Therefore, superimposed images are used throughout this study for the same reason.

In the anterior midgut, AaSeR-1 specific immunofluorescence was often found in the nucleus of some principal cells (Fig 4B). For confirmation, imaging recordings were carried out in a multi-track mode with two different channels. While the green channel (Ex. 488) was set to detect AaSeR-1 specific immunofluorescence (Fig 4C), the blue channel (Ex. 405) was recording DAPI (DNA-specific) fluorescence (Fig 4D). Co-localization analyses have unequivocally confirmed AaSeR-1 specific immunofluorescence in the nucleus. To the best of our knowledge, there are no reports of nuclear localization of GPCRs or 5-HT receptors in insects. Therefore, these findings will provide a useful milestone for reexamination of current views on a 5-HT origin and its role as a neurohormone in mosquito and insect physiology. It must be noted that this finding is not unprecedented; nuclear-localized GPCRs have been identified in species from diverse phyla, from nematodes to humans [40–43]. Data from several studies suggest that nuclear-localized GPCRs regulate specific signaling pathways with the same second messenger system associated with GPCR signaling at the plasma membrane; this includes G protein subunits [44–46], cAMP [47, 48] and IP3 [49]. Nuclear-localized GPCRs modulate gene transcription by either interacting with transcription factors [50, 51], or regulating histone acetylation [52, 53]. Whereas the majority of analyses demonstrated AaSeR-1 in the nucleus of principal cells of the anterior midgut, in a few tissues examined, AaSeR-1 specific immunofluorescence appeared in the nucleolus. While DAPI stains the nucleus quite well in blue, nucleolus usually appears unstained (Fig 4D, white arrow). Therefore, a brightfield image superimposed with green (AaSer-1 specific) and blue (DAPI—nucleus specific) fluorescence has clearly depicted AaSeR-1 in the nucleolus (Fig 4E and 4E-1). The nucleolus is of major importance for ribosome biosynthesis as well as in responses to cellular stress [54, 55]. Taken together, these results suggest 5-HT involvement in the regulation of nuclear (nucleus and nucleolus) functions. Although the physiological roles and significance of AaSeR-1 nuclear expression are currently unknown, it is important to point out that the receptor presence at different cell compartments may mediate various 5-HT effects. For instance, while Parathyroid hormone-related protein (PTHrp) binding to plasma membrane receptors suppresses cell proliferation, binding of this ligand to nuclear receptors stimulates cell proliferation [56–58].

In the posterior midgut, AaSeR-1 immunofluorescence follows the cell contours and appears to be at the plasma membrane of the principal and regenerative cells (Fig 4F and 4G). These findings will require further investigations to confirm AaSeR-1 plasma membrane localization, since the use of membrane markers is incompatible with the fixation protocol employed in this study. In addition, AaSeR-1 immunofluorescence was noted in some enteroendocrine cells (hereafter EECs) of the posterior midgut (Fig 4H). The EECs are irregular shaped cells scattered among the other cells of the midgut epithelium but predominantly found in the posterior midgut [59]. Although the role of midgut EECs in insects is not fully understood, it is known that they are the source of hormones that regulate diuresis [60], gut motility [61] and other physiological processes [59]. EECs immunoreactive to FMRFamide and small cardioactive peptide b were found earlier in the *Aedes* gut [62]. Hormones produced and secreted by EECs may mediate either systemic (60) or local effects (61). Association of AaSeR-1 with some EECs suggests that 5-HT may mediate indirect physiological effects through regulation of other hormones biosynthesis.

**Fig 5:**
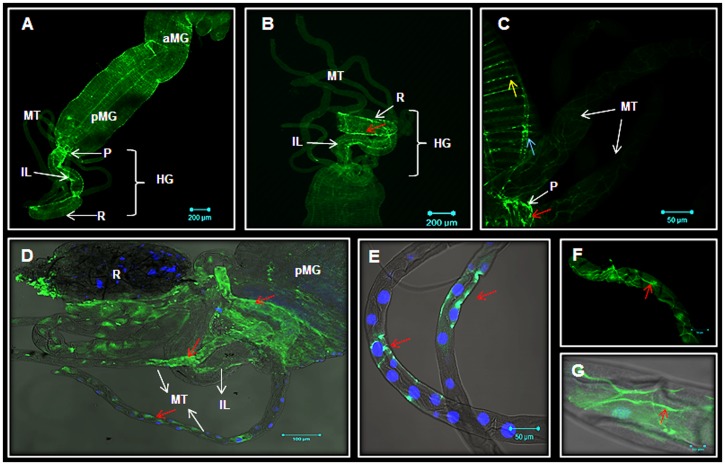
AaSer-1 expression in the visceral gut muscles and the MT of *Aedes* larvae. A low magnification image (A) is depicting AaSeR-1 specific immunofluorescence in *Aedes* gut muscles (Legend: aMG-anterior midgut, pMG-posterior midgut; MT―Malpighian tubules; HG-hindgut; P-pylorus; IL-ileum; R-rectum). AaSeR-1 is also found in the HG (B; red arrow). Higher magnification image (C) is showing AeSeR-1 expression in both circular (yellow arrow) and longitudinal (blue arrow) muscles, as well as in the pylorus area (red arrow). AeSeR-1 immunofluorescence is also found in some principal cells of the MT (D-E; red arrow). Although still under investigation, specific immunofluorescence was detected in what appears to be the tracheolar network of the MT (F-G; red arrow). Scale bars (μM) are included in each image.

Gut motility is a key physiological process enabling food passage through the alimentary canal. It is carried out by a network of visceral intrinsic muscles that innervate the larval gut [63]. This grid-like structure is comprised of circular and longitudinal muscles [63] whose function is to maintain gut integrity as well as to carry out peristalsis. AaSeR-1 specific immunofluorescence was found in both types of muscles (Fig 5A–5C) in the mid- and hindgut (pylorus, ileum and rectum), suggesting that this receptor mediates the stimulatory function of 5-HT on *Aedes* gut motility. These findings are supported by earlier reports of 5-HT effects on anterior midgut motility in *Aedes* larvae [12].

AaSeR-1 immunofluorescence (Fig 5D) appears in the MT, the primary organ for ion and fluid homeostasis. In Dipteran insects, MT are composed of principal and stellate cells [64]. Despite their simplistic cell composition, each region of the MT has its specific role in diuresis. Diuresis is initiated by secretion of ions and solutes in the distal part (the blind-ended part) of the MT into the tubule lumen, followed by water secretion [65]. This, in turn, increases the hydrostatic pressure in the tubule lumen, resulting in a downstream flow towards the proximal segment of the MT and directed to the gut [65]. Solute reabsorption (not water) takes place along the way, followed by secretion of diluted fluid. While principal cells mediate the transepithelial Na^+^ and K^+^ secretion, stellate cells are involved in Cl^-^ secretion [65]. AaSeR-1 specific immunoreactivity was observed in the principal and not in the stellate cells throughout the MT with no distinct expression patterns (Fig 5D and 5E). However, it must be pointed out that the majority of analyzed samples have exhibited AaSeR-1 specific immunoreactivity in either distal or proximal parts. These results suggest that 5-HT may mediate an array of effects in the MT through a spatial (or temporal) expression of AaSeR-1. Previous studies also pointed out that 5-HT has differential effects on the MT function in *Rhodnius*; while in the distal parts 5-HT mediates fluid secretion [66], in the proximal regions 5-HT regulates K+ uptake [67]. Current results have provided a basis for further analyses that will reexamine the role of 5-HT in mosquito diuresis, since doubts on its involvement in this process have been cast by previous studies [68]. In addition, although still under investigation, some results suggest that AaSeR-1 is part of the tracheolar system in the MT (Fig 5F and 5G).

**Fig 6:**
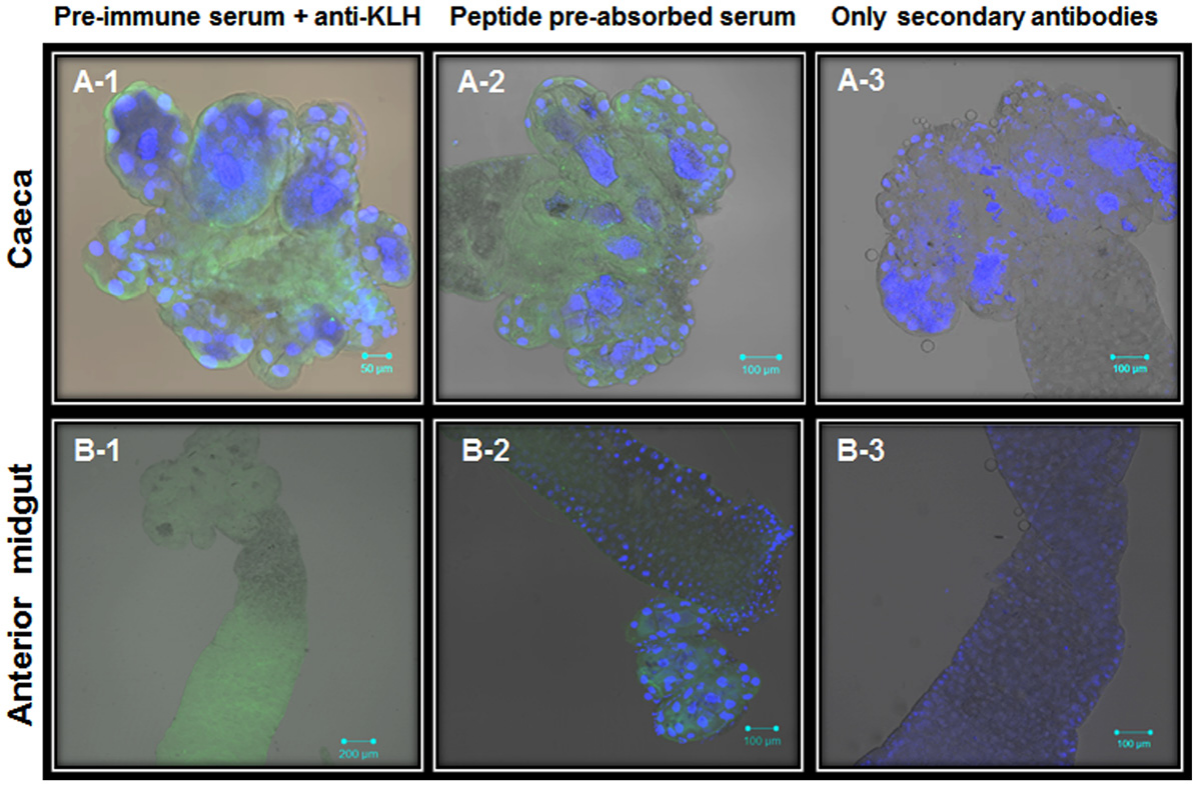
Specific AaSeR-1 immunofluorescence was not observed in the caeca and the anterior midgut of *Aedes* larvae when subjected to negative control analyses. Brightfield images of *Aedes* caeca (A) and anterior midgut (B) superimposed with green (Ex. 488) and blue fluorescence (DAPI—DNA specific, Ex. 405) were obtained from samples treated with (1) pre-immune serum (1:500) + anti-KLH antibody (1:10000), or (2) peptide pre-absorbed anti-AaSeR-1 antiserum (1:500) + anti-KLH antibody (1:10000), or (3) secondary antibodies (1:300). Lack of specific (strong) green fluorescence in the control tissues indicates that there is no cross-reactivity between negative control treatments and *Aedes* gut endogenous proteins. Scale bars (μM) are included in each image.

**Fig 7:**
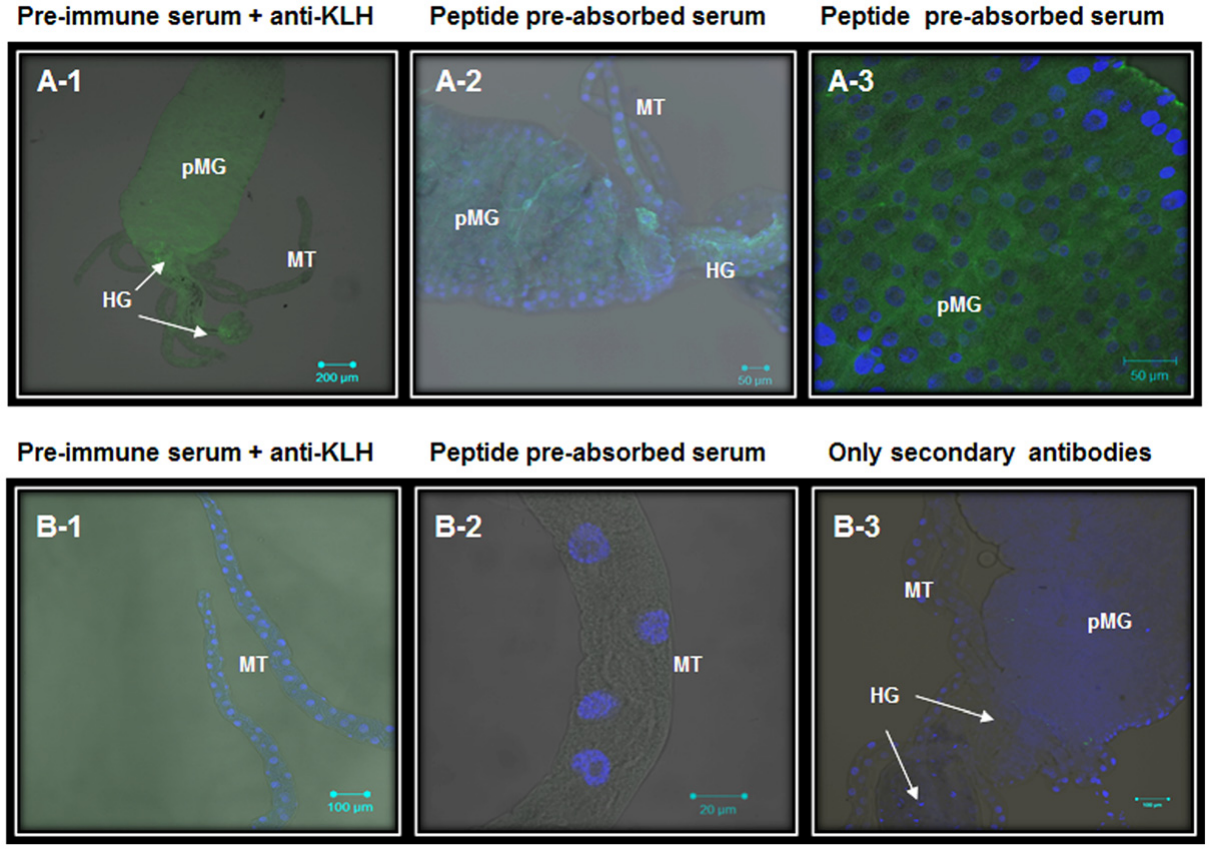
No specific expression of AaSeR-1 was observed in the posterior midgut or the hindgut of *Aedes* larvae when subjected to negative control analyses. A low magnification brightfield image of *Aedes* mid- and hindgut (A-1) superimposed with green fluorescence (Ex. 488) was obtained from a sample treated with pre-immune serum (1:500) + anti-KLH antibody (1:10000) instead of anti-AaSeR-1 antiserum. Recordings were taken under high ‘gain’ option for green fluorescence. This was carried out to show that even at high ‘gain’, the background green fluorescence is homogeneous and dull, indicating that there is no cross-reactivity between the pre-immune serum and *Aedes* gut endogenous proteins. Similar results were obtained when samples were treated with peptide pre-absorbed anti-AaSeR-1 antiserum (1:500) + anti-KLH antibody (1:10000) (A-2; A-3). The same trend was also observed for the MT when subjected to pre-immune serum+ anti-KLH antibody (B-1) or peptide pre-absorbed anti-AaSeR-1 antiserum + anti-KLH antibody (B-2). Samples incubated with secondary antibodies only (1:300) did not exhibit any specific green fluorescence as well (B-3). Nuclear staining (DAPI, Ex. 405) is shown in A-2, A-3, B-1, B-2 and B-3. Scale bars (μM) are included in each image. Legend: pMG-posterior midgut; MT—Malpighian tubules; HG-hindgut.

Understanding cell molecular mechanisms that have been under ‘nature’s research and development’ mode for millions of years is a difficult and challenging job. Therefore, it is important to emphasize that different approaches and methodologies need to be employed to ensure rigorous investigations. Future studies on AaSeR-1 localization will include different methodologies such as transmission electron microscopy, subcellular fractionation, etc. to reconfirmed current findings and possibly provide more detailed analyses on AaSeR-1 expression at the plasma membrane. However, for now, the current study has initiated the ‘learning phase’ of understanding 5-HT roles in mosquito digestion and homeostasis by employing the immunohistochemical approach to understand AaSeR-1 expression patterns. A large number of larvae (>100) have been under investigation where, in parallel to AaSeR-1 localization analyses, different sets of negative controls were carried out to determine the level of background fluorescence, including unspecific binding. None of these recordings have exhibited strong or specific immunofluorescence patterns (Figs 6 and 7), providing reasonable basis to conclude that current AaSeR-1 localization patterns are true representatives.

Overall, the study has presented a rather complex picture of AaSeR-1 functional expression patterns that suggests the following: (i) the presence of AaSeR-1 in different sections of the alimentary canal suggest its diverse roles (and thus of 5-HT) in digestion and homeostasis (at temporal and spatial level); (ii) AaSeR-1 may mediate various effects through its expression at different cell compartments; (iii) overall AaSeR-1 expression profiles indicate that 5-HT is an important neurohormone that may regulate multiple physiological processes in *Aedes* larvae.
